# Deep learning for intracranial hemorrhage detection and classification in brain CT scans: a systematic review and hybrid model approach

**DOI:** 10.3389/fdgth.2026.1703634

**Published:** 2026-04-07

**Authors:** Harshith V., Bhargavram Athray, Likhitha T. Murthy, Samyukta Joshi, Ameena Amreen Ayoob, Sai Chakith M. R., Pruthvish Reddy, Ranjith Raj, Vikram Patil, Deepak Benny, Shiva Prasad Kollur, Kasim Sakran Abass, Victor Stupin, Sushma Pradeep, Chandan Shivamallu, Ekaterina Silina

**Affiliations:** 1 Computer Science and Engineering (Artificial Intelligence and Machine Learning), Vidyavardhaka College of Engineering, Mysuru, Karnataka, India; 2 Department of Pharmacology, JSS Medical College & Hospital, JSS Academy of Higher Education & Research, Mysuru, Karnataka, India; 3 Department of Cell Biology and Molecular Genetics, SDU Academy of Higher Education & Research, Tamka, Kolar, Karnataka, India; 4 Department of Biotechnology, Acharya Institute of Technology, Bengaluru, Karnataka, India; 5 Department of Radio Diagnosis, JSS Medical College & Hospital, JSS Academy of Higher Education & Research, Mysuru, Karnataka, India; 6 School of Physical Sciences, Amrita Vishwa Vidyapeetham, Mysuru, Karnataka, India; 7 Department of Physiology, Biochemistry, and Pharmacology, College of Veterinary Medicine, University of Kirkuk, Kirkuk, Iraq; 8 Department of Hospital Surgery, Pirogov Russian National Research Medical University, Moscow, Russia; 9 Centre for Digital Health & AI, JSS Medical College, JSS Academy of Higher Education & Research, Mysuru, Karnataka, India; 10 Department of Biotechnology & Bioinformatics, School of Life Sciences, JSS Academy of Higher Education & Research, Mysuru, Karnataka, India; 11 Institute of Digital Biodesign and Modeling of Living Systems, I.M. Sechenov First Moscow State Medical University (Sechenov University), Moscow, Russia

**Keywords:** intracranial hemorrhage, convolutional neural networks (CNNs), machine learning, deep learning, computed tomography

## Abstract

**Background:**

Intracranial hemorrhage (ICH) is a life-threatening medical emergency requiring rapid and accurate diagnosis. Non-contrast computed tomography (CT) remains the primary imaging modality for detecting acute hemorrhage. In recent years, machine learning (ML) and deep learning (DL) approaches have gained increasing attention for automated detection and classification of ICH and its subtypes. This systematic review aims to consolidate and critically analyze contemporary machine learning and deep learning methodologies applied to ICH detection and classification from non-contrast CT scans.

**Methods:**

A comprehensive review of published studies was conducted focusing on ML and DL models developed for identifying ICH and its subtypes, including epidural, subdural, intraparenchymal, intraventricular, and subarachnoid hemorrhages. The reviewed techniques encompass conventional convolutional neural networks (CNNs), three-dimensional CNNs, hybrid and ensemble frameworks, and emerging transformer-based architectures. Preprocessing strategies such as Hounsfield Unit windowing, skull stripping, and data augmentation were examined. Additionally, explainable artificial intelligence (XAI) approaches, including Grad-CAM, were evaluated for enhancing model interpretability.

**Results:**

Recent studies demonstrate promising diagnostic performance across multiple deep learning architectures, with improved sensitivity and specificity for subtype classification. Hybrid and transformer-based models show enhanced feature representation capabilities. Preprocessing techniques and explainability methods contribute significantly to model robustness and clinical interpretability.

**Conclusion:**

Machine learning and deep learning models exhibit substantial potential in automated ICH detection and classification from non-contrast CT scans. However, challenges remain regarding generalizability, dataset heterogeneity, and clinical validation. Future research should emphasize large-scale multi-center validation, model interpretability, and integration into real-world clinical workflows to enable effective translation into routine neuroimaging practice.

## 1 Introduction

ICH is a severe neurologic emergency with high morbidity and mortality if not diagnosed early and managed appropriately. In fact, although ICH accounts for only a small percentage of strokes, it causes more than 50% of the mortality related to stroke; thus, early diagnosis is critical (1). The possibility of deterioration is higher within the first 24 h, and early detection may improve the outcome. The initial imaging technique to evaluate for suspected ICH is non-contrast computed tomography (CT), which has a rapid throughput, is widely available, and is very sensitive for detecting acute hemorrhage. CT is still the first-line modality in emergencies and for trauma patients when patients present with acute alterations in consciousness or they are taking anticoagulants, even though MRI is very helpful when assessing the age of a hemorrhage or change over time. Determining if a patient needs monitoring, follow-up imaging, or urgent intervention relies on detecting possibly small hemorrhages. Unfortunately, visual assessment of CT images can be a complicated and time-consuming process, especially in busy clinical environments where minor or subtle deviations may be missed in the absence of a decision-support mechanism (2).

In order to surmount these limitations, the focus of recent research has been to develop various machine learning and deep learning techniques for ICH detection and subtype classification automatically. The common general procedures followed are preprocessing, feature extraction, and classification, while the most prevalent metrics used for their performance evaluation comprise accuracy, sensitivity, specificity, AUC, precision, recall, and *F*1-score (3). Deep CNN architectures have shown very strong performances in a wide variety of medical imaging applications, while a number of works related to hemorrhage subtype classification used 3D CNNs, hybrid models which combine deep learning with classical machine learning approaches, as well as more recent transformer-based approaches (4).

Despite there being several survey papers reviewing DL-based methods, many have limitations. Some of them lack comparisons of datasets in a structured way or of preprocessing steps or strategies for evaluation, while others focus solely on deep learning models and completely disregard conventional ML techniques (5). Moreover, several critical issues still exist that affect the robustness and generalisation ability of automated systems, such as class imbalance, inconsistent expert labelling, and variability in CT acquisition settings (1). This review aims to consolidate existing machine learning and deep learning approaches for the detection and classification of ICH from CT scans. We summarise commonly used models, datasets and preprocessing techniques; compare different evaluation measures; and outline strengths and limitations arising in previous work. We also identify open challenges in research and outline future directions that may help bring automated ICH detection closer towards practical deployment within clinical settings (6).

CT remains the preferred modality for initial evaluation in cases of intracerebral hemorrhage, both traumatic and non-traumatic. MRI has a vital role to play in the age-based differentiation of hemorrhage; however, CT is indispensable for rapid diagnosis, especially in acute settings like trauma or anticoagulant use. The detection of even subtle hemorrhages is crucial for clinical management. Manual interpretation is difficult to perform, time-consuming, and susceptible to oversight by less experienced practitioners (7).

**Figure 1:**
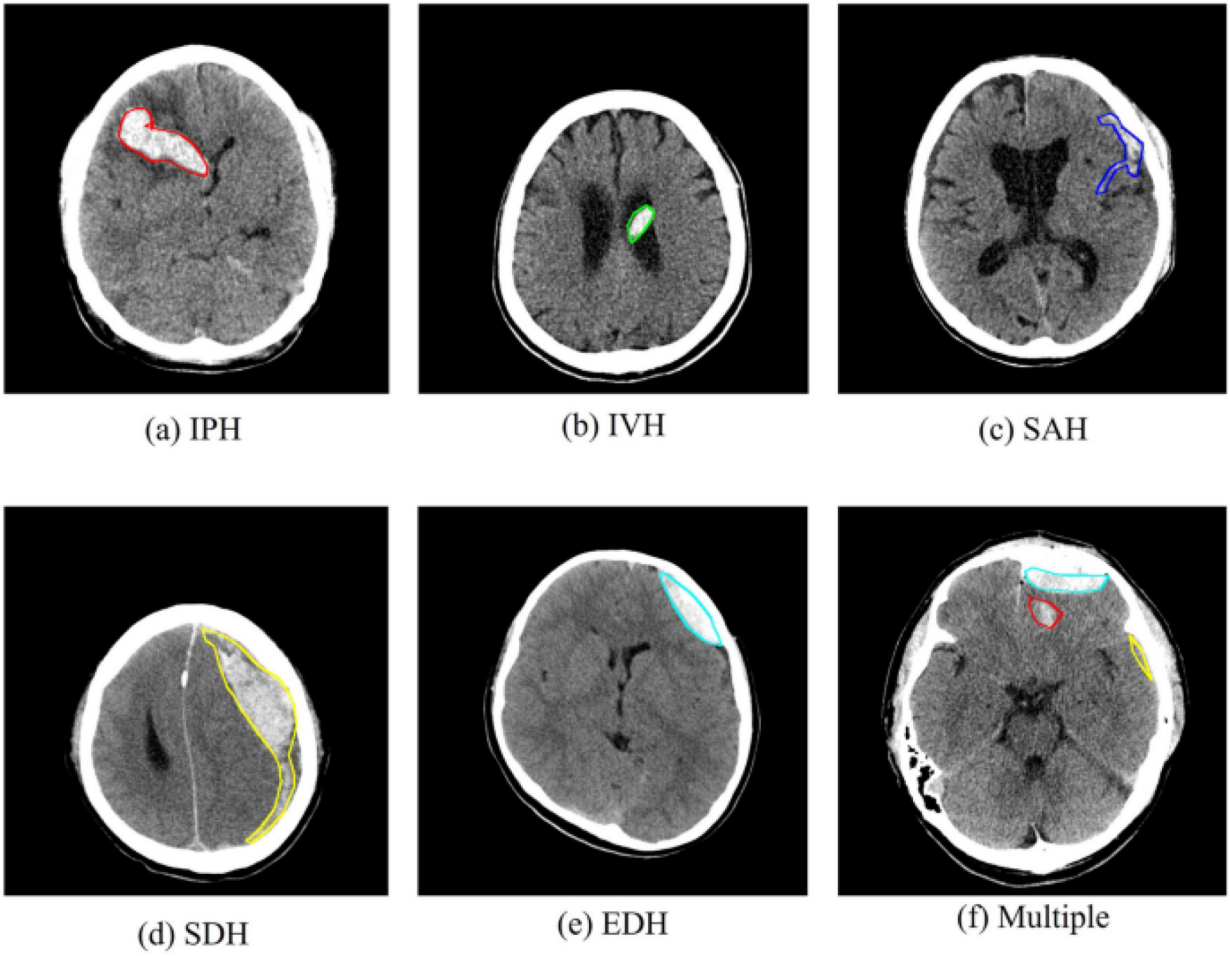
Illustrative examples of different types of brain hemorrhages in CT scans. The hemorrhagic regions are highlighted in red. **(a)** IPH, **(b)** IVH, **(c)** SAH, **(d)** SDH, **(e)** EDH, **(f)** Multiple. Reproduced from “Data examples; (a) example of intraparenchymal hemorrhage (IPH); (b) example of intraventricular hemorrhage (IVH); (c) example of subarachnoid hemorrhage (SAH); (d) example of subdural hemorrhage (SDH); (e) example of epidural hemorrhage (EDH); (f) CT images with one or more cerebral hemorrhagic lesions (the image include IPH, SDH, and EDH)” by Zhegao Piao, Yeong Hyeon Gu, Hailin Jin and Seong Joon Yoo, licensed under CC BY 4.0.

Techniques relying on both classical machine learning and deep learning have recently been proposed with the aim of improving speed and accuracy in brain hemorrhage detection and classification from CT scans. Several recent surveys present these trends as reviews of methodologies, goals, preprocessing pipelines, and dataset characteristics over 15 years. Some are focused on conventional machine learning techniques; others are based on deep learning–assisted approaches or hybrid pipelines that combine both. Comparisons between single-slice and multi-slice models, patch-based vs. full-image strategies, and region-localisation vs. full-segmentation frameworks have also been explored. These reviews collectively indicate the rapid pace of evolution in automated ICH analysis while highlighting inconsistencies in methodology, evaluation criteria, and dataset use across studies (8) (Figure 1).

Despite growing interest, significant challenges remain in multi-class ICH classification due to variations in dataset size, limited diversity, and the rarity of accurately labelled medical images. Deep learning approaches continue to expand across healthcare applications, and both machine learning and deep learning methods have become important to handle large-scale medical image data (10). However, subtle differences in preprocessing, windowing strategies, and augmentation techniques, along with varying architectural choices, often lead to significant performance differences, making comparisons across studies difficult.

Brain imaging is essential in the neurological work-up. Although MRI helps in estimating the age of hemorrhage, CT is very important in quickly and confidently identifying acute bleeding, especially in trauma or patients with anticoagulant therapy. The detection of even minimal hemorrhages becomes imperative for distinguishing appropriate management pathways. Manual reading of CT studies requires a very high level of expertise; most practising radiologists have to contend with fatigue, time constraints, and unequal experience (11). These factors tend to detract from diagnostic precision, and studies illustrate subtle hemorrhages may be missed with manual readings, particularly by the less experienced clinical reader.

The advancement of DL further enhanced diagnostic performance for many medical imaging tasks, often surpassing human-level performance in certain pattern recognition tasks. CNNs have risen to prominence in image understanding tasks across a wide range of applications, and have seen rapid adoption. Many articles have identified challenges in selecting suitable CNN architectures, preprocessing datasets, and extracting features (12). Despite these successes, misunderstandings about the complexity of DL have hindered its adoption among some medical practitioners, prompting other efforts to simplify DL-based medical imaging workflows.

Brain hemorrhage is still one of the most severe neurological emergencies, related to different aetiologies like trauma, hypertension, vascular abnormalities, venous thrombosis, and aneurysms (1). Though it comprises only 10%–15% of all strokes, it is responsible for more than half of stroke-related deaths (12). Rapid detection is vital because early intervention dramatically improves patient survival and reduces long-term morbidity. At the same time, the wide variability in clinical presentations and imaging appearances makes consistent diagnosis challenging.

These factors indicate an increasing demand for a review that integrates findings from studies using classical ML, deep learning, hybrid, and ensemble-based models; that discusses preprocessing and dataset differences; performs a comparative analysis of evaluation metrics; and emphasises existing challenges and future research opportunities. This paper aims to address these needs by providing a structured analysis of the state of the art, highlighting methodological trends, and outlining promising directions for further research in automated detection and classification of ICH using CT imaging.

## 2 Methodology

### 2.1 Study selection and PRISMA flow summary

A thorough literature search was performed across databases such as PubMed, Scopus, Web of Science, IEEE Xplore, ScienceDirect, Google Scholar, as well as grey-literature sources like arXiv and ResearchGate. The search yielded 2,146 records, plus an additional 112 from manual searches and reference checks (13). After removing duplicates, 1,873 records were screened by title and abstract. From these, 561 full-text articles were evaluated for eligibility based on criteria focusing on deep learning and machine learning methods for detecting intracranial hemorrhage via CT scans.

A total of 482 full-text studies were excluded for reasons including non-deep-learning focus, non-CT modalities, incomplete methodological description, lack of validated datasets, or being review/editorial papers (14). Ultimately, 79 studies met the inclusion criteria for the qualitative synthesis, and 34 studies were included in the quantitative comparison tables. The PRISMA 2020 flow summary describing this selection process is presented in the Table 1 below.

**Table 1:** The PRISMA 2020 flow summary describing the selection process.

Stage	Description	Number of records
Identification	Records identified through database searching (PubMed, Scopus, IEEE, Web of Science, ScienceDirect, Google Scholar)	*n* = 2,146
	Additional records identified from other sources (reference lists, arXiv, ResearchGate, grey literature)	*n* = 112
	Records after duplicates removed	*n* = 1,873
Screening	Titles and abstracts screened	*n* = 1,873
	Records excluded (irrelevant outcomes, non-ICH imaging, non-CT studies, insufficient methods)	*n* = 1,312
Eligibility	Full-text articles assessed for eligibility	*n* = 561
	Full-text articles excluded with reasons: • Not ML/DL-based ICH detection (*n* = 153) • Review/editorial/letter papers (*n* = 102) • Incomplete metrics or missing dataset validation (*n* = 87) • Inadequate CT imaging quality/artifact-heavy scans (*n* = 66) • Not CT-based ICH studies (*n* = 74)	*n* = 482
Included	Studies included in qualitative synthesis	*n* = 79
	Studies included in quantitative comparison/summary tables	*n* = 34

### 2.2 Literature survey

Wang et al. designed a custom deep learning model for the segmentation and classification of acute ICH from the CT scans (15). This way, a model based on a 2D CNN in combination with sequence models was trained on the 2019 RSNA Brain CT Hemorrhage Challenge dataset and showed good performance with AUCs up to 0.996. It was further externally iteratively validated employing datasets including PhysioNet-ICH and CQ500. The paper shows the possibility of applying ensemble learning with CNNs such as SE-Resnext101 and Densenet169, in addition to the Grad-CAM technique for model interpretability. The proposed model helps radiologists to diagnose ICH accurately and in less time.

Tharek et al. (16) proposed an intracranial Hemorrhage detection technique in CT scans employing CNNs for deep learning. The authors employed a dataset of 200 de-identified CT scans involving Hemorrhage and non-Hemorrhage conditions. As for the measurement of model performance, sensitivities of 96.94%, specificity of 93.14%, and the accuracy of 95% were reached. This paper points towards the value of early diagnosis in lowering case fatality because of cerebral Hemorrhage, especially subtypes including EDH, SDH and SAH. However, as the authors pointed out, CT perk also limits its ability to recognize minor Hemorrhages, so it's best not to eliminate the radiologist supervision.

For example, Singh et al. (17) developed a shallow 3D CNN for detection of different Hemorrhages such as SAH, IPH, SDH and IVH. They developed a feature-enhancing procedure for standardisation of corrected 3D volumetric CT scans to enhance detection. Built on the CQ500 dataset, the model has shown high *F*1 scores in the detection of Hemorrhage, including SAH at 0.96 and IVH at 0.99. The paper also investigated various CNN architectures such as VggNet, ResNet etc and finally found that the shallow 3D CNN was the most suitable for the purpose of Hemorrhage assessment in CT scans.

The authors by Sage and Badura (18) proposed an effective hybrid deep learning system for ICH detection in CT images using double-branch CNN with SVM and Random Forest classifiers. The study utilised a large dataset of 372,556 CT slices and achieved very high metrics for identifying Hemorrhage subtypes, such as IVH and IPH. Majority of the results centred on the accurate feature extraction and the more refined spatial-intensity feature selection for higher detection rates and overall making the paper a potential candidate for clinical applications.

The HU region in brain CT images with the ICH Babu and Brindha (19) presented a complex hybrid model, DenseNet 121 and LSTM. For feature extraction, DenseNet was used and for feature classification, LSTM was used. Performance comparison of their model showed that it has an accuracy of 97.50%, precision of 97.00%, recall of 95.99%, and *F*1-score of 96.33%. Analysing the results, the paper showed that the integrated approach of CNN and LSTM increases the detection rates, eliminated the overfitting problem, and propose a more powerful model for ICH classification.

Ahmed et al. proposed a deep learning model based on EfficientDet with Grad-CAM and ResNet for ICH detection from the CT scan (20). The developed model attained an accuracy of 92.7% in the classification of patients with sepsis and a ROC AUC of 0.978. The study focused on applying EfficientDet for effective output with regard to precision for viable resource usage. To bridge the interpretability information gap, Grad-CAM was employed to provide visual illustrations for the model's choices. This approach is especially helpful if interpretability of the AI decisions made is important and especially when used in clinical applications (Table 2).

**Table 2:** Survey 1 conducted for the study.

Author(s)	Title	Purpose	Algorithms used	Evaluation metric
Wang et al. (15)	A deep learning algorithm for automatic detection and classification of acute intracranial hemorrhages in head CT scans	To develop a deep learning algorithm for detecting and classifying ICH subtypes	Ensemble CNNs (SE-ResNext101, DenseNet121, DenseNet169), Grad-CAM	AUC, log loss
Tharek et al. (16)	Intracranial hemorrhage detection in CT scan using deep learning	To detect hemorrhage types and improve early diagnosis	Convolutional neural networks (CNN)	Accuracy, precision, sensitivity, specificity, *F*1 score
Singh et al. (17)	Shallow 3D CNN for detecting acute brain hemorrhage from medical imaging sensors	To propose a shallow 3D CNN for improved classification of hemorrhage types	Shallow 3D CNN, 3D VggNet, ResNet	*F*1 score, precision, recall
Sage and Badura (18)	Intracranial hemorrhage detection in head CT using double-branch convolutional neural network, support vector machine, and random forest	To detect ICH subtypes using a hybrid model combining deep learning and traditional classifiers	Double-branch CNN (ResNet-50). random forest, support vector machine	Accuracy (ACC), sensitivity (TPR), specificity (TNR), *F*1 score
Babц and Brindha (19)	Deep learning fusion for intracranial hemorrhage classification in brain CT imaging	To classify ICH subtypes using hybrid DenseNet121 and LSTM models	DenseNet121, LSTM	Accuracy, precision, recall, *F*1 score
Ahmed et al. (20)	Exploring deep learning and machine learning approaches for brain hemorrhage detection	To use EfficientDet with interpretability tools for accurate ICH detection	EfficientDet, ResNet, Grad- CAM	Accuracy, ROC AUC, sensitivity
Cortés-Ferre et al. (21)	Deep learning applied to intracranial hemorrhage detection	To classify CT slices for hemorrhage presence and provide patient- level diagnoses with visual explanations	EfficientDet for classification, Grad-CAM for visual explanations	Miss rate, true positive rate (TPR)
Umapathy et al. (22)	Automated computer-aided detection and classification of intracranial hemorrhage using ensemble deep learning techniques	To enhance ICH detection and classification through ensemble techniques	SE-ResNet with LSTM, Grad-CAM	Accuracy, *F*1 score, interpretability (Grad- CAM)
Nizarudeen and Shunmugavel (23)	Multi-Layer ResNet-DenseNet architecture in consort with the XgBoost classifier for intracranial hemorrhage (ICH) subtype detection and classification	ICH subtype detection and classification	ResNet-DenseNet with XgBoost classifier	Accuracy, precision, recall, *F*1 score, ROC curve
Pradeep et al. (24)	Automatic detection and segmentation of brain hemorrhage based on improved U-Net	Brain hemorrhage detection and segmentation	Improved U-Net architecture with different backbones: DenseNet-121, ResNet-50, and MobileNet-V2	Accuracy (>99%), precision, recall, *F*1 score
Mushtaq et al. (25)	BHCNet: neural network-based brain hemorrhage classification using head CT scan	Brain hemorrhage classification	Neural Network (BHCNet)	Accuracy, precision, recall, specificity, and *F*1-score.

In the D'Angelo et al. study, a new deep learning pipeline was tested for the detection and classification of various types of ICH and midline shifts from NCCT scans of TBI patients (24). Their findings indicated strong diagnostic performance with 91.24% accuracy in the detection of hemorrhage and even more accurate (98.54%) classification of hemorrhage type, while cutting down diagnosis time by a large margin compared to radiologists. These results underscore the increasing significance of deep learning solutions in optimizing diagnostic processes, especially in urgent emergency situations, and validate further incorporation of AI-based tools into clinical workflows.

By improving the conventional U-Net architecture, Dinesh Kumar et al. present a novel method for identifying and classifying brain hemorrhages in CT scans in their 2024 study, “Automatic Detection and Segmentation of Brain Hemorrhage based on Improved U-Net Model.” To improve the U-Net framework's feature extraction capabilities, the authors integrate three potent pre-trained backbones: DenseNet-121, ResNet-50, and MobileNet-V2. The enhanced models show excellent segmentation accuracy, up to 99%, when tested on a publicly accessible Kaggle dataset that includes both hemorrhagic and non-hemorrhagic CT images (26). This study demonstrates how encoder-decoder architectures and transfer learning can be used to create precise and effective automated neuroimaging diagnostic tools.

Cortés-Ferre et al. (21) settled the assessment of the performance of the AIDOC AI Algorithm in the case of CT scan ICH. The research included 4,946 non-contrasted CT scan cut-outs collected in 18 hospitals and sought to find a comparison between the results given by AI and the radiologist. The scenario was different in that they claimed 12.2% of more cases, and radiologists missed cases by 12.4%. Even with these discrepancies, the paper endorsed the utilization of AI in the radiologist's practice in view of the fact that this AI can work in off hours and in emergencies and can make the work of triage faster and more clinically efficient.

Umapathy et al. (22) presented an article in which they tested the potential of ensemble deep learning methods for CT scans for ICH, its recognition, localization, and classification. With the combination of SE-ResNeXT and LSTM, the authors obtained an appealing *F*-score of 0.97 and an accuracy of 99.79%. Effectively, their model categorized four different types of hemorrhage (EDH, IVH, SAH, IPH, SDH). The study used datasets RSNA with the CQ500 for training and diagnostic windows. In addition, the use of Grad-CAM in the model further enhanced confidence in AI-integrated decisions. This meant that the hybrid deep learning approach was appropriate for clinical use.

### 2.3 Overview of techniques

The majority of deep learning research for ICH detection has centered on CNNs, specifically in two dimensions: two-dimensional (2D) and three-dimensional (3D). For instance, Wang et al. employed a 2D CNN coupled with sequence models to detect ICH with precision and classify its subtypes. Likewise, Tharek et al. utilized CNN-based architectures for the analysis of CT scan data and attained high precision in hemorrhage detection (15, 16).

In the past few years, hybrid models have come into the spotlight for enhancing performance. Hybrid models integrate CNNs with other machine learning methods including LSTM networks and conventional classifiers such as Random Forest (RF) and Support Vector Machine (SVM). For example, Babu and Brindha (19) integrated Dense Convolutional Network (DenseNet-121) with LSTM to extract both spatial and temporal features from 3D CT scan volumes, leading to enhanced classification of hemorrhage types (Table 3). Other research, for example, Sage and Badura (18) and Singh et al. (17), investigated the same hybrid strategies that combine CNNs with machine learning classifiers in order to increase the detection of ICH subtypes like Subdural Hemorrhage (SDH), Epidural Hemorrhage (EDH), Subarachnoid Hemorrhage (SAH), and Intraventricular Hemorrhage (IVH).

**Table 3:** Comparative analysis of selected deep learning models for intracerebral hemorrhage (ICH) classification.

Attribute	Study 1	Study 2	Study 3
Authors/year	OzNet et al., 2023	ResNet-Inception + LGBM (2023)	DenseNet + Bayesian Optimization (2023)
Dataset (samples)	Public (815 slices)	Public (500 scans)	Private (1,074,271 slices)
Objective	4-class	6-class	4-class
Model	OzNet + Fully Connected Network	ResNet101 + InceptionV4 + LGBM	DenseNet with Bayesian Optimization
Validation	10-fold	Single-fold	Single-fold
Performance metrics	AUC = 0.99, Acc = 0.93	Acc = 0.95, Se = 0.95, Sp = 0.94	Acc = 0.948, Pr = 0.854

Table 2 compares three 2023 studies, detailing the datasets, classification objectives, models, validation methods, and performance metrics such as AUC (Area Under the Curve), Acc (Accuracy), Se (Sensitivity), Sp (Specificity), and Pr (Precision).

Another essential facet of deep learning in medical imaging is model interpretability. Techniques such as Gradient-weighted Class Activation Mapping (Grad-CAM) and saliency maps are commonly employed to visualize the regions of a CT image that the model finds most relevant in making its prediction. This visual feedback can be validated by radiologists to ensure the model's decision-making, thereby making the AI system more reliable in a clinical environment. Both Wang et al. (15) and Babu and Brindha (19) utilized Grad-CAM successfully to promote understandability and transparency in their model predictions.

### 2.4 Dataset utilization

All the studies reviewed in this work use publicly available datasets for training, validation, and testing deep learning models. The three most widely used datasets in this field are the Radiological Society of North America (RSNA) Brain Computed Tomography (CT) Hemorrhage Challenge, CQ500, and PhysioNet-ICH. These datasets are diverse and large, consisting of CT scans that are tagged with various types of Intracranial Hemorrhage (ICH), such as Subdural Hemorrhage (SDH), Epidural Hemorrhage (EDH), Subarachnoid Hemorrhage (SAH), Intraparenchymal Hemorrhage (IPH), and Intraventricular Hemorrhage (IVH) as in Table 4.

**Table 4:** Distribution of labeled samples for various hemorrhage types and non-hemorrhagic (normal) cases in the CT scan dataset.

SDH	EDH	IPH	IVH	SAH	None
24,912	1,482	13,666	19,026	18,353	3,16,082

For instance, the RSNA Brain CT Hemorrhage Challenge dataset, as used by Wang et al. (15), comprises more than 25,000 labeled CT scans, offering a solid basis for model training and model performance evaluation. Such datasets are generally divided into three subsets—training, validation, and testing—to test a model's capacity to generalize to new, unseen instances.

Research such as Umapathy et al. utilized the RSNA and CQ500 datasets to train and validate ensemble learning models, where various model architectures are fused together (22). This enhances robustness and guarantees that the model works optimally under diverse clinical conditions.

It should be added that in the majority of the hemorrhage annotations in the considered datasets, the labels are produced either by human expert radiologists manually or through semi-automatic software. Even though manual annotation provides high-quality results, they take time and depend on the human factor. Alternatively, auto- or semi-auto-labeling will add a little uncertainty to the process, particularly when the ground truth quality is not scrutinously checked (23). This emphasizes that for effective training of models, one needs to have high-quality annotations as well as well-constructed data.

### 2.5 Preprocessing

**Figure 2:**
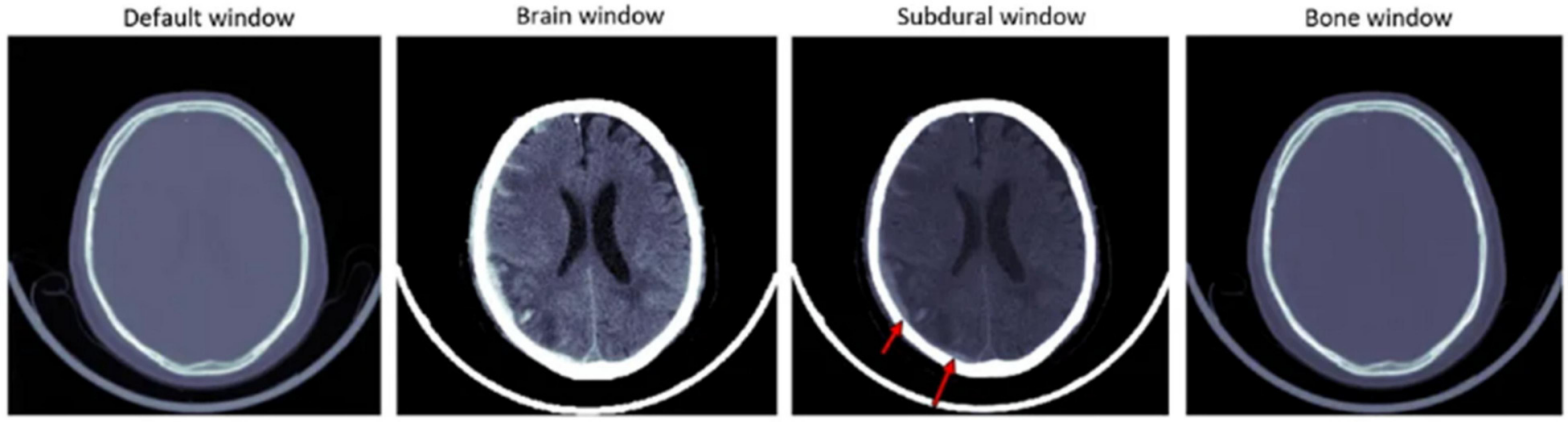
CT scan images under different window settings: default window, brain window, subdural window, and bone window. Images derived from the RSNA Intracranial Hemorrhage CT Dataset (https://www.rsna.org/artificial-intelligence/ai-image-challenge).

To improve the quality and stability of the input data, a series of preprocessing techniques have been introduced prior to the delivery of the CT images to the deep learning models. Image normalization is a ubiquitous step in research that results in the azimuthal standardization of CT image intensity and model robustness to scan quality variations (24). Tharek et al. (16) normalize the images via techniques that force the images to be similar, thus minimizing the possibility that results from different scanners will differ. Data augmentation is another important method to address challenges like imbalanced datasets, where some hemorrhage types are underrepresented (25). Rotation, flipping, and cropping are some techniques that have been applied to enlarge the number of samples artificially and to improve the generalization of the model. Wang et al. (15) and Babu and Brindha (19) use these augmentation methods to address the data imbalance and train the model so that it works for all types of hemorrhages (Figure 2).

Windowing, as in CT, is applied to a subset of studies to select specific Hounsfield Unit (HU) ranges that represent regions of Hemorrhage. Singh et al. (17) extended this approach to better discriminate brain hemorrhages by separating the target structures from the CT scans to improve the hemorrhage detection accuracy (27).

Several of the reviewed studies include external validation to ensure their findings apply beyond their training datasets. For example, the model developed by Wang et al. (15) was first trained on the RSNA Brain CT Hemorrhage Challenge dataset. It was then validated on independent datasets like PhysioNet-ICH and CQ500. This method improves the model's strength and reliability in different clinical situations. Including this step is essential for any future models intended for use in real-world clinical settings (15, 28).

### 2.6 Model architectures

In this survey, Grad-CAM is shown as a popular method for improving model interpretability by visualizing attention areas in CT images. Although this review does not include original model training or evaluation, we emphasize that studies like Wang et al. (15) and Umapathy et al. (22) have reported qualitative validation of Grad-CAM outputs by experienced radiologists. This highlights the role of clinical experts in confirming AI decisions, particularly for future use in real-world diagnostic environments (29).

In contrast, Singh et al. (17) investigated shallow 3D CNNs for hemorrhage detection and found that shallow 3D CNNs can achieve good performance with limited data. This illustrates one of the benefits of shallower networks in cases of data scarcity as opposed to deeper networks that are otherwise more likely to overshoot small datasets (30–34). Hybrid architectures that integrate the CNNs and classical machine learning techniques are also widely used in the literature. Sage and Badura (18) proposes a double-branch CNN coupled with Random Forest and SVM, which computes various feature extraction schemes and classification methods to enhance the quality of the models (Table 5). In a similar way, Babu and Brindha (19) fuses DenseNet121 with LSTM networks to improve the extraction of features from the 3D volumetric datasets and compute the classification accuracy as a result (35).

**Table 5:** Comparison of Various hemorrhage detection techniques.

Author(s)	Dataset used	ICH subtypes	Key contributions
Wang et al. (15)	2019-RSNA brain CT hemorrhage challenge dataset, PhysioNet-ICH, CQ500	EDH, IPH, IVH, SAH, SDH	Developed an ensemble deep learning algorithm with CNNs and sequence models; ranked first in RSNA challenge; introduced Grad-CAM for interpretability.
Tharek et al. (16)	200 anonymized CT scans from public datasets	EDH, SDH, SAH, contusion hemorrhage	Achieved high accuracy and sensitivity using CNN for hemorrhage detection; emphasized early detection for better clinical outcomes.
Singh et al. (17)	CQ500 dataset	SAH, IPH, SDH, IVH	Proposed a shallow 3D CNN architecture; achieved high *F*1 scores for individual hemorrhage types; highlighted preprocessing for 3D volumetric CT scans.
Sage and Badura (18)	Dataset of 372,556 CT slices from 9,997 patients	SDH, EDH, IPH, IVH, SAH	Introduced a hybrid model combining CNN, SVM, and random forest; focused on feature extraction and classification to enhance subtype detection accuracy.
Babц and Brindha (19)	Public datasets and augmentation techniques	EPD, ITP, ITV, SDH, SAH	Combined DenseNet121 and LSTM for feature extraction and classification: achieved high accuracy and reduced overfitting using data augmentation strategies.
Ahmed et al. (20)	RSNA intracranial hemorrhage competition, clinical datasets	ICH, non-ICH	Used EfficientDet with Grad-CAM for efficient detection and interpretability; emphasized minimizing false positives while maintaining high AUC scores.
Cortés-Ferre et al. (21)	4,946 non-contrast HCT scans from 18 hospitals	ICH	Evaluated Al tool AIDOC; identified more ICH cases than radiologists with explainable outputs using Grad-CAM; highlighted challenges in artifact handling.
Umapathy et al. (22)	RSNA brain CT hemorrhage challenge, CQ500	EPD, IPH, IVH, SDH, SAH	Proposed ensemble deep learning with SE-ResNext and LSTM: achieved near-perfect accuracy: used Grad-CAM for visualizations and enhanced decision-making.

In addition, methods, e.g., those presented in Umapathy et al. (22) to enhance the detection accuracy and robustness, integrate the architecture of several CNNs such as SE-ResNext101 and DenseNet169 (Table 6). The performance of ensemble models has been proved to be better, especially for difficult detection tasks, such as hemorrhage localization (36, 37).

**Table 6:** Binary classification performance metrics (normal vs. subarachnoid hemorrhage) for different deep learning models, including sensitivity, specificity, precision, *F*1 score, accuracy, and AUC.

Metric	S3DCNN	B3DCNN	VggNet1
Sensitivity	0.95	0.91	0.93
Specificity	1.00	0.93	0.94
Precision	1.00	0.94	0.95
*F*1 score	0.96	0.92	0.94
Accuracy	0.96	0.92	0.93
AUC	0.981	0.961	0.921

#### 2.6.1 Transformer-based architectures and entropy-aware fusion strategies

For medical image analysis, Vision Transformer or ViT based models represent recent AI advancements. They present important gains in performance vs. usual CNNs (38). As a promising approach, the Pyramid Vision Transformer (PVT) combines CNNs' local feature extraction strengths with Transformers' long-range dependency modeling. PVT obtains contextual information at multiple scales, especially helping Hemorrhage region detection in CT scans with irregular, heterogeneous shapes (4).

Integrating PVT with the explainable AI (XAI) techniques, the powerful diagnostic model was one that was proposed by that outstanding study that was titled “Entropy-aware Fuzzy Integral Fusion of SHAP-Selected Transformer Embeddings for Interpretable Intracranial Hemorrhage Classification” (39). SHAP values highlight important features from transformer embeddings; this isolation improves interpretability while also reducing model complexity (40).

With the model, an entropy-aware fuzzy integral fusion mechanism improves the decision robustness. Regarding confidence levels (entropy), this technique adaptively weights each CT slice's contribution (41). Influence which is greater is given to slices with a high certainty. Slices bearing ambiguous signals, however, are downweighted. Being especially important in real-world clinical settings that have noisy or partial data, this approach minimises the noise, balances the decision risk, and stabilises the overall predictions (42).

### 2.7 Evaluation metrics

In order to assess the performance of the models, the researchers employ a number of metrics, with a major emphasis on classification performance. Accuracy, precision, sensitivity, specificity, and *F*1 score are commonly reported metrics across all studies. For example, Babu and Brindha (19) shows accuracies with high precision, recall, and *F*1 score of 97.50% in diagnosing ICH subtypes. Umapathy et al. (22) report an accuracy of 99.79% and an *F*1 score of 0.97, with a focus on the strength of their ensemble models (40).

Besides classification metrics, Area Under the Curve (AUC) scores are also commonly reported, e.g., as in RSNA Challenge competitions. For example, In Papers Wang et al. (15), the AUC achieves 0.996 for ICH subtypes and demonstrates a fairly high discrimination capability for the model. Localization quantifications like Grad-CAM and other localization tools (Intersection over Union, IoU) are also applied to measure the model's performance in correctly localizing hemorrhage points on CT (41). This is particularly important in clinical applications where it is sometimes possible to use an accurate localization to make design decisions faster (42).

### 2.8 Clinical integration challenges and best practices

One of the most critical barriers to the integrating of Artificial Intelligence (AI) into clinical workflows for medical imaging is found especially within Picture Archiving and Communication Systems (PACS) (43). PACS is the backbone, as it stores, retrieves, and distributes radiological images across healthcare facilities (44). For AI to add real clinical value, AI models must be interoperable with PACS and Electronic Health Record (EHR) systems. This interoperability will ensure minimal disruption to the existing workflow.

For AI integration, rapid secure access is needed for CT scan imaging data. Every single second counts in cases of emergency or trauma, so then this access is important. Abilities for predicting immediately matter. Near-real-time prediction capabilities are also key (45). AI models must remain strong for dealing with suboptimal scan conditions common in emergency settings, such as motion artifacts, low-resolution images, or incomplete datasets.

Human elements matter just as much as technical issues. The AI decisions have to be trusted by the clinicians that must understand just how those decisions are made. Visualization tools like Gradient-weighted Class Activation Mapping (Grad-CAM) offer interpretability, though they do not fully satisfy clinicians' transparency demand (46). AI tools, therefore, must be accompanied by intuitive user interfaces that align with the decision-making processes of radiologists as well as neurologists. With interactive feedback mechanisms included for clinician verification, editing, or AI-generated output correction, clinicians learn together and trust AI systems more (47).

Ultimately, it is necessary to address technological and human-centric challenges for AI to be effectively and ethically implemented in clinical practice. Data scientists as well as software engineers along with clinicians plus hospital administrators must collaborate in order to implement a clinical integration strategy (48). Their collaboration will ensure AI solutions are safe practical as well as scalable for successful use.

## 3 Results and discussion

### 3.1 Preprocessing techniques in automated ICH detection

The prevalence of preprocessing techniques in the reviewed studies indicates their significance in improving the quality of CT scans. Hounsfield Unit windowing continues to be a popular choice in the field due to its capability to highlight tissue-specific contrasts. The prevalence of data augmentation methods indicates a need to address class imbalance and small dataset sizes, which are still prevalent in medical imaging research. The differences in preprocessing techniques among studies indicate a lack of standardization, which could be a contributing factor to the variability in performance reported in the literature.

### 3.2 Deep learning architectures for hemorrhage detection

The prevalence of convolutional neural network-based architectures indicates their efficacy in extracting spatial information from CT scans. Architectures such as ResNet and DenseNet are commonly identified in the literature, possibly because of their ability to extract hierarchical features while addressing vanishing gradient problems. The use of three-dimensional CNNs in some studies indicates an attempt to incorporate volumetric information; however, their higher computational complexity and requirements may hinder their applicability in clinical settings.

### 3.3 Hybrid and ensemble modeling approaches

To overcome the drawbacks of single-model designs, hybrid and ensemble modeling approaches have been investigated. By integrating different feature representation modalities or decision outputs, these models attempt to enhance robustness to hemorrhage subtypes. Although some studies have demonstrated enhanced classification consistency through these techniques, the added complexity and training overhead may serve as a hindrance to their practical application in clinical settings. Moreover, the lack of standardized assessment protocols makes it difficult to compare these hybrid techniques.

### 3.4 Explainability and interpretability considerations

The incorporation of explainability techniques, especially those based on Grad-CAM, indicates an emerging need for model interpretability in clinical decision support systems. Through visual explanation maps, clinicians can interpret whether the model focus corresponds to anatomically valid areas, thus facilitating enhanced interpretability. Although these techniques are extensively employed, they remain largely qualitative, and their clinical validation is still in its infancy. Future studies should aim to develop standardized frameworks for the assessment of explainability in automated ICH detection systems.

### 3.5 Quantitative performance summary and meta-analysis considerations

The quantitative performance evaluation metrics used in the studies considered in this review are primarily accuracy, area under the receiver operating characteristic curve (AUC), sensitivity, specificity, Dice similarity coefficient, and *F*1-score. However, the differences in evaluation methodologies, dataset partitioning, and reporting metrics among the studies considered in this review cause large methodological heterogeneities. Consequently, meta-analysis of effect sizes may lead to incorrect inferences. Future studies should aim at standardized benchmarking and reporting to facilitate meta-analytic evaluation of automated intracranial hemorrhage detection systems.

## 4 Limitations and challenges

Despite the encouraging outcomes, there exist certain limitations. The problem of data imbalance is widespread, especially for rare classes of hemorrhages such as Epidural Hematoma (EDH). While this may be better with the application of data augmentation methods, this is not a foolproof way of overcoming the situation. Likewise, the narrow range and small amounts of openly accessible repositories, for example, RSNA and CQ500, may also limit their application in models developed for them on different patients imaging systems (49).

Moreover, another issue that arises is that false positives and false negatives can occur due to several reasons. One such reason includes, but is not limited to, distortions internal to the CT/jpeg scan, vector motion blur, or even static features such as calcification (50). The authors of studies such as Ahmed et al. (20) point out such misclassification errors, thus stressing the importance of effective preprocessing and artifact correction strategies. Furthermore, there is the problem that arises from the excessive reliance on high-quality labeled datasets, as this involves a tedious process of manual labeling by skilled radiologists and suffers from inter-observer differences (51).

Although there are techniques like Grad-CAM that have advanced model interpretability, this too is still a work in progress. Health care practitioners require a more profound understanding of how the models evaluate and assign significance to the various components of the imaging data that inform risky decisions (52). In addition, the application of these models into clinical practice also involves some issues to do with the speed at which they can be executed and how they can be added to the established systems; aspects that are scanty in the majority of the sources.

## 5 Future work

The future work in Intracranial Hemorrhage Automated detection is aimed at resolving the existing deficiencies and optimally increasing the reliability and usability of all AI models in use within the clinical setup. One of the major aspects is the growing of their diversity in content. Hence, the creation of larger and richer datasets that contain less common types of hemorrhages and demographic, imaging, and scanner type variations is fundamental in the development of more efficient and transferable models (53).

Another, yet a very promising direction, would be the enhanced preprocessing techniques. For instance, acquiring novel imaging processes, such as computer-aided design, inverting x-ray machines' ceramics, and coupling them with advanced image denoising algorithms could work miracles in optimizing image quality (54). This could help to minimize the number of false detections as well as missed detections and therefore, uphold the quality of predictions (55).

A promising area for future research involves combining multimodal data sources, such as patient demographics, clinical histories, and laboratory results, with imaging data. Including these diverse inputs can improve model context, reduce diagnostic uncertainty, and strengthen the reliability and clinical value of deep learning systems in ICH detection (56).

## 6 Conclusion

The present survey surveys recent advances in deep learning for the detection, classification, and segmentation of automated intracranial hemorrhage (ICH) in CT imaging. The progression away from the baseline CNN pipelines and pre-trained approaches has provided the field with hybrid models such as CNN, LSTM, CNN, SVM, and SE-ResNeXt + LSTM structures. In addition, recent work has utilized novel frameworks like Pyramid Vision Transformers, double-branch segmentation networks, and 3D CNN models to acquire spatial and context for improved subtype classification capabilities. While many of the studies showed strong and robust diagnostic capabilities for automated ICH detection or classification, the quality of the model performance came from including a precedence of studies that had an overall reporting of accuracy values above 92%, and some above 95%, for subtypes like IVH and IPH. This reinforces that the ICH models have the potential to mitigate missed diagnoses in clinical settings where time is of the essence. The overall workflow can incorporate improved pre-processing steps like skull stripping, intensity normalisation, and multi-window CT enhancement for improved diagnosis. Tools like Grad-CAM can also improve model interpretability for implementation in practice. Although there have been significant advancements through deep learning for ICH identification, there are multiple actionable challenges, such as lack of annotated labelled data, class imbalance, nonstandard imaging protocols, and limited generalisation across institutions that demonstrate a clear pathway crudely delineating the clinical development of scalable and adaptable systems that can be implemented within an institutional clinical workflow. This example reinforces the value of hybrid spatial–sequential learning approaches and underscores the practical relevance of evidence-driven model design. Despite promising progress, challenges such as data imbalance, limited dataset diversity, and cross-institutional generalizability remain, indicating clear directions for future research toward scalable and clinically deployable AI systems for intracranial hemorrhage diagnosis. This review paper provided a comprehensive approach towards recent and current methods, but note that there are gaps in our ability to leverage the methodological advances from deep learning studies to the limitations of data in the midst of crossing boundaries that focuses on the upcoming research opportunities directions in expanding the ICH diagnosis and resultant treatments.

## Data Availability

The original contributions presented in the study are included in the article/Supplementary Material, further inquiries can be directed to the corresponding authors.
